# Localized Hotspot Management: Hand-Held Phage Aerosols as a Complementary Strategy for Carbapenem-Resistant *Acinetobacter baumannii* Infection Control in Healthcare Settings

**DOI:** 10.3390/antibiotics15010038

**Published:** 2026-01-01

**Authors:** Yao-Song Lin, Li-Kuang Chen, Hsiu-Yen Chien, Ruei-Sen Jiang, Chun-Chieh Tseng

**Affiliations:** 1 Department and Graduate Institute of Public Health, Tzu Chi University, Hualien 97004, Taiwan; 109324109@gms.tcu.edu.tw (Y.-S.L.); chienhy@gms.tcu.edu.tw (H.-Y.C.); 111324103@gms.tcu.edu.tw (R.-S.J.); 2 Institute of Medical Sciences, College of Medicine, Tzu Chi University, Hualien 97004, Taiwan; lkc@tzuchi.com.tw; 3 Department of Clinical Pathology, Buddhist Tzu Chi General Hospital, Hualien 97002, Taiwan

**Keywords:** bacteriophage, aerosol, hand-held sprayer, surface decontamination, carbapenem-resistant *A. baumannii*

## Abstract

**Background**: Carbapenem-resistant *Acinetobacter baumannii* (CRAB) remains a major challenge in healthcare settings due to its persistence on inanimate surfaces and resistance to conventional cleaning methods. Bacteriophages (phages) represent a promising biocontrol option owing to their high specificity and lytic activity. **Methods**: This study evaluated the use of a personal hand-held vibrating mesh nebulizer (VMN) as a rapid and localized delivery platform for phage aerosols. Using two lytic phages (ϕ2, Podovirus; ϕ11, Myovirus), we assessed phage stability under different storage conditions, viability during VMN operation, and surface decontamination efficacy under varying spray parameters. **Results**: In saline, both phages showed optimal long-term stability at 4 °C, whereas storage at −20 °C resulted in a progressive reduction in infectivity exceeding 3 logs over the storage period. VMN aerosolization did not compromise viability. A 3 min spray achieved >99.9% surface reduction: ϕ2 was effective at 1 × 10^7^ PFU/mL, whereas ϕ11 required 1 × 10^8^ PFU/mL. Importantly, residual ϕ2 activity persisted for at least 24 h, preventing detectable recolonization under the assay conditions, while ϕ11 protection was limited to 6 h. **Conclusions**: These findings establish the hand-held sprayer as a practical, low-cost, and flexible approach to deliver viable phage aerosols, providing an effective complement to large-scale disinfection systems and offering a targeted strategy to enhance infection control in healthcare environments.

## 1. Introduction

Healthcare-associated infections (HAIs), particularly those caused by carbapenem-resistant *Acinetobacter baumannii* (CRAB), represent a significant global public health challenge, especially in intensive care units (ICUs) [1]. *A. baumannii* is notorious for its ability to persist on inanimate surfaces for extended periods, contributing to its widespread transmission within healthcare environments [2]. Current conventional cleaning and disinfection methods often struggle to eradicate these resilient pathogens from environmental surfaces completely [3], leaving potential reservoirs for patient infection. The persistent challenge of achieving comprehensive environmental decontamination underscores the critical need for novel and complementary infection control strategies.

Bacteriophages (phages), natural bacterial viruses, are emerging as a promising alternative or adjuvant in the fight against antibiotic-resistant bacteria, owing to their high host specificity and potent lytic activity [4]. Phages are typically safe for eukaryotic cells and can multiply at infection sites, providing ongoing biocontrol unlike chemical disinfectants [5]. These characteristics make phages attractive candidates for environmental decontamination in sensitive healthcare settings.

Our previous research has explored the application of phage-containing aerosols in real-world hospital environments [6,7,8]. Large-scale aerosol generators were utilized in hospital ICUs, effectively reducing CRAB acquisition rates and decreasing carbapenem resistance percentages among *A. baumannii* isolates [6,7,8]. This approach proved to be a comprehensive and time-saving method for general ward decontamination. However, while efficient for broad area disinfection, these large-scale systems may be less practical for immediate, on-demand, localized decontamination of specific, frequently touched surfaces or small, hard-to-reach areas within a clinical setting. The primary focus of these earlier studies was on patient infection outcomes. Thus, the direct evaluation of surface decontamination efficacy and the operational flexibility for localized applications were not fully explored.

To address this critical gap and enhance the flexibility and precision of phage-based environmental control, the current study investigates the efficacy of phage aerosols generated by a personal hand-held sprayer for targeted and localized surface decontamination. This portable device enables rapid, on-site responses to localized contamination without requiring large equipment or patient relocation. This research was conducted in a laboratory chamber setting, systematically evaluating the precise parameters influencing phage aerosol performance and decontamination efficacy against CRAB.

Specifically, this study aims to: (1) evaluate the stability of selected phages in various storage conditions and within the hand-held sprayer, (2) assess the distribution of phage aerosols within an experimental chamber, (3) determine the optimal conditions (phage concentration, spray time) for surface decontamination of CRAB using the hand-held sprayer, and (4) investigate the persistence of phage infectivity on surfaces after application. By establishing these optimal parameters, this study could provide a valuable tool for proactive localized infection control, offering a practical and flexible approach to complement existing large-scale decontamination protocols. The findings are expected to contribute to improved patient safety and help mitigate the significant disease burden associated with CRAB in healthcare environments.

## 2. Results

### 2.1. Aerosol Characteristics of the Hand-Held Sprayer

**Figure 1:**
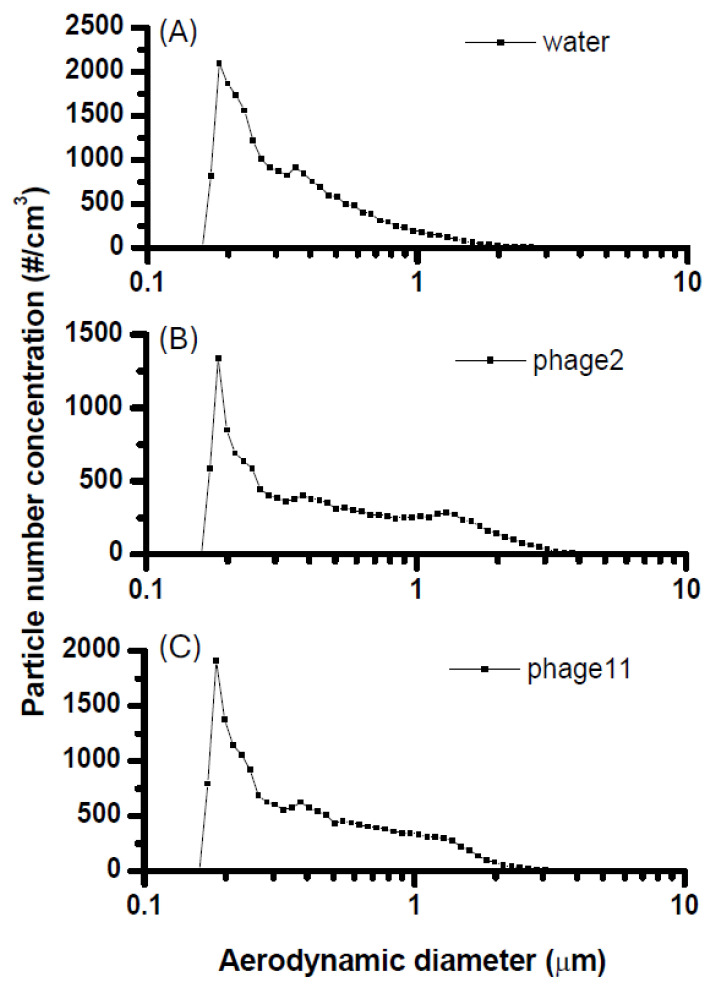
Aerosolized particle size distributions generated by the personal hand-held sprayer. (**A**) sterile water, (**B**) phage ϕ2 suspension (10^9^ PFU/mL), and (**C**) phage ϕ11 suspension (10^9^ PFU/mL). Particle number concentrations (#/cm^3^) were measured across aerodynamic diameters from 0.1 to 10 μm.

The aerosolized particle size distribution measured during operation of the personal hand-held sprayer is shown in Figure 1. In all conditions, a dominant submicron peak was consistently detected at ~0.18 μm, with a secondary rise near 0.35 μm. These modes are likely attributable to background ultrafine particles present in the chamber rather than primary droplets generated by the sprayer. When phage suspensions at 10^9^ PFU/mL were nebulized, ϕ2 aerosols showed additional minor peaks at ~0.38 μm and ~1.3 μm, while ϕ11 aerosols displayed corresponding modes at ~0.38 μm and ~1.2 μm.

### 2.2. Phage Stability Under Various Storage Conditions

The long-term stability of phages ϕ2 and ϕ11 was evaluated over 300 days at three temperatures (−20 °C, 4 °C, and 25 °C) in two aqueous media (sterile water and 0.9% normal saline). The initial phage concentration for all experiments was approximately 10^9^ PFU/mL. For ϕ2 in sterile water (Figure S1A), storage at 4 °C provided the highest stability, with a final titer of 8.17 × 10^7^ PFU/mL after 300 days. At 25 °C, moderate stability was observed, while freezing at −20 °C resulted in substantial losses over time. For ϕ2 in normal saline (Figure S1B), refrigerated storage again supported long-term stability, with a final titer of 1.0 × 10^7^ PFU/mL after 300 days. At 25 °C, ϕ2 showed greater resilience, retaining 7.05 × 10^7^ PFU/mL, but viability dropped sharply at −20 °C, reaching only 1.50 × 10^5^ PFU/mL (a ~4-log reduction).

For ϕ11 in sterile water (Figure S1C), refrigeration preserved viability better than other conditions. Phage titers gradually declined, reaching 2.2 × 10^6^ PFU/mL at 25 °C and 4.5 × 10^6^ PFU/mL at −20 °C after 300 days. For ϕ11 in normal saline (Figure S1D), refrigerated storage was the most effective, with titers maintained at 1.04 × 10^8^ PFU/mL and only a 1 log reduction after 300 days. By contrast, freezing at −20 °C was highly detrimental, with infectivity dropping below the detection limit within 80 days.

### 2.3. Phage Stability Within the Hand-Held Sprayer During Operation

To assess the impact of the aerosolization process on phage viability, the stability of phages ϕ2 and ϕ11 was monitored during a 10 min continuous operation of a hand-held sprayer (Figure S2). The experiments were conducted using initial phage concentrations ranging from 10^7^ to 10^9^ PFU/mL. Phage ϕ2 demonstrated exceptional stability, with its titers remaining virtually unchanged across all tested concentrations (Figure S2A). For instance, a suspension with an initial titer of 2.16 × 10^9^ PFU/mL measured 2.17 × 10^9^ PFU/mL after 10 min, indicating no significant loss of infectivity during operation.

Phage ϕ11 also retained high levels of activity, although a concentration-dependent effect was observed (Figure S2B). At a high initial titer of 7.83 × 10^9^ PFU/mL, the concentration only decreased slightly to 6.5 × 10^9^ PFU/mL, corresponding to a minimal loss of less than 0.1 logs. However, at the lowest initial concentration of 1.43 × 10^7^ PFU/mL, a more notable reduction to 3.67 × 10^6^ PFU/mL was measured (a 0.6-log decrease). Overall, these findings indicate that the mechanical stress of aerosolization within the hand-held sprayer does not substantially compromise phage viability, particularly at high titers (≥10^8^ PFU/mL), supporting its suitability as an effective device for phage application.

### 2.4. Aerosol Distribution and Viability in Air

**Figure 2:**
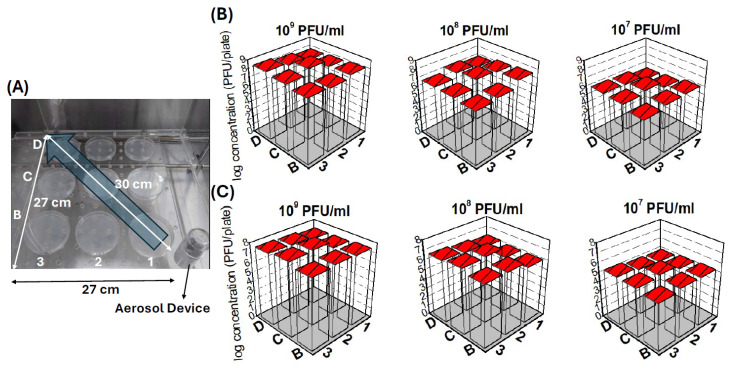
Spatial distribution of aerosolized phages ϕ2 and ϕ11. (**A**) Experimental setup showing the placement of the aerosol device (bottom right) and the arrangement of nine agar collection plates in a 3 × 3 grid. The *x*-axis (positions 1–3) represents increasing distance from the sprayer, the *y*-axis (rows B–D) represents the lateral position, and the blue arrow indicates the spray direction. (**B**) Deposition of ϕ2 aerosols and (**C**) deposition of ϕ11 aerosols following 1 min spraying at three initial titers (10^9^, 10^8^, and 10^7^ PFU/mL). The *z*-axis in each 3D plot represents the recovered phage concentration (PFU/plate). Position 2C corresponds to the central location directly in front of the sprayer, aligning with the main aerosol trajectory shown in (**A**).

The spatial distribution and viability of phage aerosols generated by the hand-held sprayer were characterized by collecting aerosolized phages on gelatin agar plates positioned at various locations. The sampling grid and resulting phage deposition after a 1 min spray are illustrated in Figure 2.

The aerosol distribution was highly focused rather than diffuse. For both phages tested, the peak concentration of viable phages was consistently recovered at the central position, 2C (the middle point directly in front of the sprayer; Figure 2A). When a 10^9^ PFU/mL suspension of ϕ2 was aerosolized, the mean recovery at position 2C was 5.52 × 10^8^ PFU/plate (Figure 2B). Statistical analysis confirmed this centrally peaked distribution, revealing that the titer at 2C was significantly higher than at all other eight positions (*p* < 0.0125).

A similar focused distribution pattern was observed for ϕ11 (Figure 2C). From an initial 10^9^ PFU/mL suspension, the maximum recovery of 8.78 × 10^7^ PFU/plate was also recorded at position 2C. The statistical results further supported a primary deposition axis; for both phages, titers at positions along the presumed direct spray path (e.g., 1B, 2C, and 3D) were significantly higher than those at peripheral locations such as 1D and 3B (*p* < 0.05). Collectively, these findings demonstrate that the sprayer generates a directed aerosol stream, effectively delivering a high concentration of viable phages along a primary, central axis with limited lateral dispersion.

### 2.5. Surface Decontamination Efficacy Against CRAB

**Figure 3:**
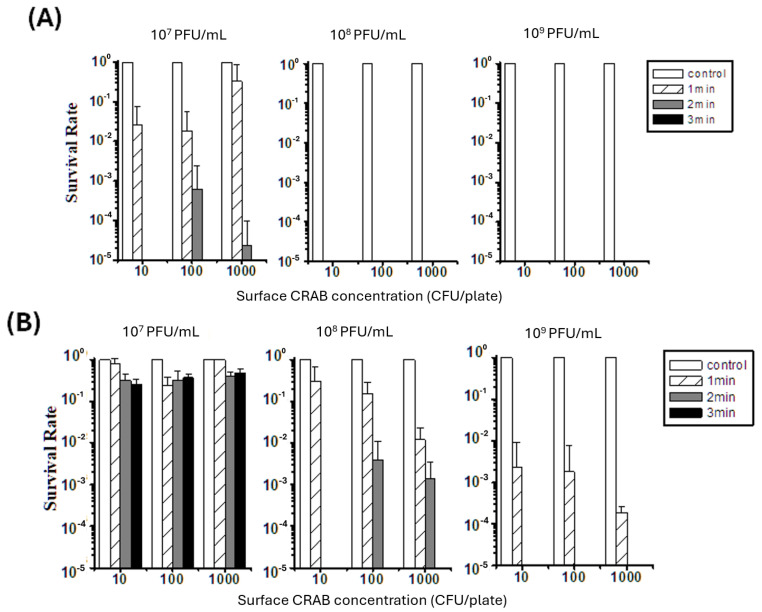
Surface decontamination efficacy of aerosolized phages ϕ2 (**A**) and ϕ11 (**B**) against CRAB. Survival rates of CRAB (*y*-axis, log scale) are shown after exposure to phage aerosols under different conditions. The *x*-axis indicates the initial CRAB surface concentration (10^1^–10^3^ CFU/plate). Each panel represents a phage suspension at 10^7^, 10^8^, or 10^9^ PFU/mL. Bars correspond to control and spraying durations of 1, 2, or 3 min, as indicated in the legend. Data represents the mean survival rates calculated from nine spatial replicates (*n* = 9) arranged in a 3 × 3 grid within the exposure chamber. Error bars represent standard deviations. Absence of bars indicates survival rates were below the detection limit.

Supported by the high phage stability confirmed during VMN operation (see Section 2.3 and Figure S2), the bactericidal efficacy of aerosolized phages ϕ2 and ϕ11 against surface-loaded CRAB was quantified in Figure 3. The experiments assessed the impact of varying phage concentrations, initial CRAB surface densities (10^1^ to 10^3^ CFU/plate), and spray durations (1, 2, or 3 min). Both phages demonstrated a clear dose- and time-dependent bactericidal activity. Phage ϕ2 exhibited high potency; at concentrations of 10^8^ and 10^9^ PFU/mL, no viable CRAB colonies were detected (0% survival), irrespective of the initial bacterial load or spray duration (Figure 3A). Even at a lower concentration of 10^7^ PFU/mL, increasing the spray duration to 3 min was sufficient to reduce CRAB survival to below the limit of detection for loads up to 10^3^ CFU/plate, a significant improvement compared to the 33.34% survival observed after a 1 min spray (*p* = 0.042).

Phage ϕ11 also showed efficacy but required a higher concentration and longer exposure time (Figure 3B). At 10^7^ PFU/mL, ϕ11 only partially reduced CRAB. However, when the concentration was increased to 10^8^ PFU/mL, a 3 min spray resulted in 0% CRAB survival across all tested bacterial densities, a statistically significant improvement over a 1 min spray (*p* < 0.001). In summary, the conditions required to reduce CRAB survival to below the detection limit were identified as a 3 min spray with 10^7^ PFU/mL for ϕ2, and a 3 min spray with a higher concentration of 10^8^ PFU/mL for ϕ11.

### 2.6. Persistence of Phage Infectivity on Decontaminated Surfaces

**Figure 4:**
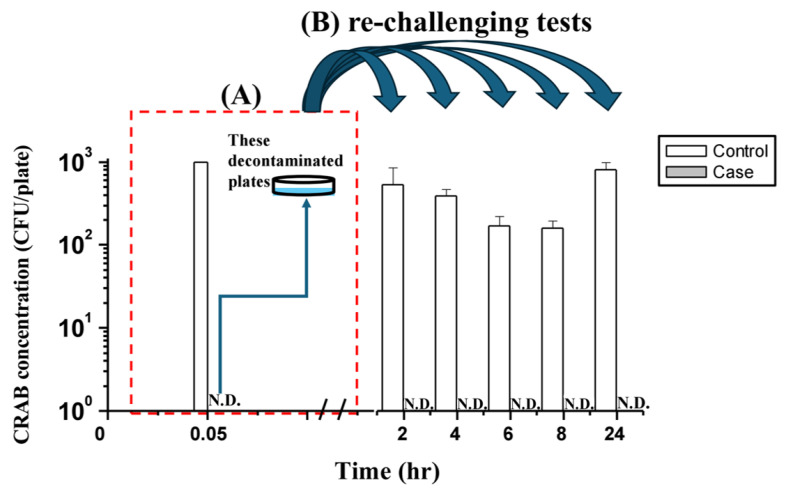
Residual surface activity of phage ϕ2 against CRAB following decontamination and re-challenging. (**A**) Initial decontamination of CRAB-contaminated surfaces (10^3^ CFU/plate) after spraying with phage ϕ2 at 10^7^ PFU/mL for 3 min (0.05 h). (**B**) Re-challenging tests performed by exposing the decontaminated plates to CRAB aerosols after resting for 2–24 h. The *y*-axis indicates CRAB concentration (CFU/plate, log scale), and the *x*-axis indicates resting time before re-challenge. Control bars represent untreated plates, while case bars represent phage-treated plates. The detection limit was 0.33 CFU/plate, defined as the presence of a single colony across three replicate plates. Values below this threshold were recorded as non-detectable (N.D.). The curved arrows illustrate the transfer of the decontaminated plates from the initial treatment (**A**) to the re-challenging tests (**B**).

The residual bactericidal activity of phages on treated surfaces was evaluated by re-challenging the surfaces with CRAB aerosols (10^2^ CFU/plate) at various time points post-decontamination (Figure 4). The initial decontamination was carried out under the optimal conditions previously identified for each phage (Figure 4). For phage ϕ2, a suspension at 10^7^ PFU/mL was sprayed for 3 min against a surface inoculated with 10^3^ CFU/plate of CRAB, after which no colonies were detectable, indicating that bacterial survival had fallen below the detection limit. These decontaminated plates were left to rest in the exposure chamber for 2, 4, 6, 8, and up to 24 h before re-challenging tests. At all these time points, CRAB remained undetectable following aerosol exposure, demonstrating that ϕ2 retained sufficient infectivity to suppress surface recolonization for at least 24 h. Two-way ANOVA performed on log-transformed data (with N.D. assigned as 0.165 CFU/plate) confirmed a highly significant main effect for the phage treatment (F(1, 20) = 8813.48, *p* < 0.001). No significant interaction between treatment and time was observed (F(4, 20) = 2.686, *p* = 0.061), indicating stable and consistent residual efficacy over the 24 h period.

**Figure 5:**
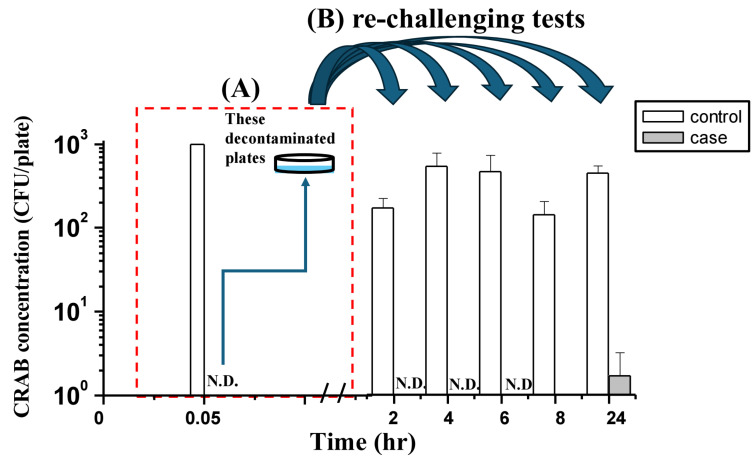
Residual surface activity of phage ϕ11 against CRAB following decontamination and re-challenging. (**A**) Initial decontamination of CRAB-contaminated surfaces (10^3^ CFU/plate) after spraying with phage ϕ11 at 10^8^ PFU/mL for 3 min (0.05 h). (**B**) Re-challenging tests performed by exposing the decontaminated plates to CRAB aerosols after resting for 2–24 h. The *y*-axis indicates CRAB concentration (CFU/plate, log scale), and the *x*-axis indicates resting time before re-challenge. Control bars represent untreated plates, while case bars represent phage-treated plates. The detection limit was 0.33 CFU/plate, defined as the presence of a single colony across three replicate plates. Values below this threshold were recorded as non-detectable (N.D.). The curved arrows illustrate the transfer of the decontaminated plates from the initial treatment (**A**) to the re-challenging tests (**B**).

In contrast, surfaces treated with phage ϕ11 exhibited a more transient protective effect (Figure 5). Following an initial decontamination with 10^8^ PFU/mL of ϕ11 for 3 min against surfaces inoculated with 10^3^ CFU/plate of CRAB, no colonies were detectable at 0.05 h, confirming that the treatment reduced bacterial survival below the detection limit. These decontaminated plates were then subjected to re-challenging after resting for different durations. Residual activity remained effective for up to 6 h, during which no CRAB colonies were detected upon re-challenge. However, after an 8 h resting period, a small number of colonies (0.33 ± 0.58 CFU/plate; *n* = 3) became visible, though the values were so low that they were nearly indistinguishable in Figure 5. By 24 h, survival further increased (1.67 ± 1.53 CFU/plate; *n* = 3). Two-way ANOVA revealed a significant interaction between phage treatment and time (F(4, 20) = 3.915, *p* = 0.017), indicating a decline in residual potency over time. Bacterial presence was observed at eight hours and 24 h. However, the overall phage-treated group remained significantly lower than the control at all time points (*p* < 0.001). These findings indicate that, under the tested conditions, ϕ2 can maintain residual surface activity for at least 24 h. In contrast, the adequate residual protection of ϕ11 is limited to approximately 6 h.

### 2.7. Surface Phage Concentration Dynamics

**Figure 6:**
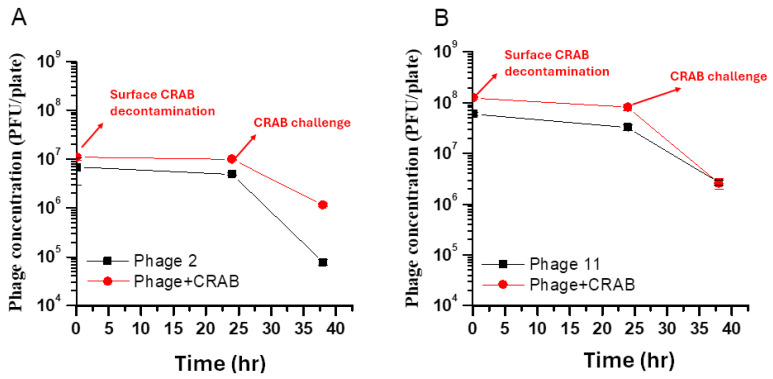
Persistence of surface phages with or without CRAB exposure. Agar plates were sprayed with phage ϕ2 (10^7^ PFU/mL, 3 min; (**A**)) or ϕ11 (10^8^ PFU/mL, 3 min; (**B**)). Test group (red circles) received a surface decontamination step at 0 h by applying CRAB at 10^3^ CFU/plate concurrently with phage spraying; control group (black squares) received phage only. Phage titers were measured at 0 h, at 24 h, and 14 h later (38 h total). At 24 h, test plates were challenged with CRAB aerosols generated from a 10^7^ CFU/mL suspension (10^2^ CFU/plate by sedimentation) and then incubated for 14 h before the final measurement. Data are PFU/plate on a logarithmic scale.

Two-way ANOVA revealed significant main effects for both time and treatment, as well as a significant interaction between these factors for both phage types (*p* < 0.001). For ϕ2 (Figure 6A), the interaction effect was highly significant (F(2, 12) = 28.70, *p* < 0.001), indicating that the impact of CRAB exposure on phage persistence varied over time. Post hoc analysis using Tukey’s HSD showed that ϕ2 concentrations remained stable between 0 h and 24 h (*p* = 0.627), maintaining a level of approximately 10^7^ PFU/plate. Following the CRAB aerosol challenge at 24 h, the test group retained 1.16 × 10^6^ PFU/plate at 38 h, which was significantly higher than the natural decay observed in the control group (7.75 × 10^4^ PFU/plate; *p* < 0.001).

For ϕ11 (Figure 6B), Two-way ANOVA confirmed a highly significant interaction between CRAB treatment and time (F(2, 12) = 19.69, *p* < 0.001), along with a significant main effect of the CRAB treatment group (F(1, 12) = 64.39, *p* < 0.001). The phage titers in the test group (2.58 × 10^6^ PFU/plate) and the control group (2.67 × 10^6^ PFU/plate) reached a similar order of magnitude by 38 h. However, compared to the distinct gaps observed at 0 h and 24 h, the survival curves of ϕ11 exhibited a marked convergence following the CRAB aerosol challenge. This suggests that ϕ11 initially maintains a higher surface concentration in the presence of CRAB, but its long-term persistence dynamics at the terminal stage are more closely aligned with its natural decay rather than the host-mediated amplification observed for ϕ2.

## 3. Discussion

The escalating CRAB burden in healthcare-associated infections underscores the urgent need for novel control strategies. In Taiwan, *A. baumannii* ranked as the seventh most common ICU pathogen in early 2024 [9], with persistently high resistance and considerable clinical and economic impact [1]. Previous studies have indicated that CRAB isolates are generally more susceptible to phage infection than non-resistant strains (84% vs. 56.5%) [10], suggesting that phage-based approaches could effectively target these prevalent resistant populations. Building on this rationale, the present study evaluated the practical feasibility of phage-based decontamination using a personal hand-held sprayer, focusing on aerosol stability, spatial distribution, and bactericidal efficacy. This aerosol-based approach represents a promising and complementary tool for infection control in clinical environments.

Although the handheld vibrating-mesh nebulizer predominantly produces large, visible droplets along the primary jet, submicron particles (0.18–1.3 µm) were nonetheless detected in peripheral zones of the chamber—plausibly formed via rapid droplet evaporation under moderate humidity (~55% RH), leaving behind suspended droplet nuclei [11,12]. The close overlap of particle-size distributions between phage suspensions and pure water further suggests that the presence of phages exerts minimal influence on atomization, with particle formation primarily governed by bulk fluid properties such as surface tension and viscosity [13].

Our results confirm that 4 °C is the optimal storage condition, consistent with previous studies [14], supporting the requirement for maintaining a ready-to-use phage stock. In contrast, the rapid loss of infectivity at −20 °C, even in a chemically compatible medium such as physiological saline, highlights the dominant role of freezing-associated physical stress [15,16]. Ice crystal formation can mechanically damage phage particles. At the same time, localized increases in solute concentration during freezing impose osmotic stress, both of which accelerate structural disruption [16].

In the phage stability assays, phages were intentionally tested in simple aqueous solutions without cryoprotectants, reflecting the anticipated use scenario in which only a short interval exists between preparation and application. For a surface decontamination product, additives such as glycerol or other cryoprotectants are not desirable because they may alter aerosol characteristics or leave residues on treated surfaces [13], ultimately reducing decontamination performance. Together, these findings provide a clear rationale for recommending storage at 4 °C to preserve maximum efficacy and stability, thereby establishing guidelines for proper handling and reliable performance of the phage-based decontaminant.

Nebulization can expose phages to substantial mechanical stress, with the extent of damage depending on device design. Jet nebulizers are particularly disruptive; Astudillo et al. (2018) reported that up to 83% of aerosolized phages showed structural breakage under electron microscopy [17]. In contrast, vibrating mesh nebulizers impose less shear stress, with only ~50% structural alteration and minimal impact on infectivity [17]. Kutter et al. (2021) observed no significant loss of phage activity, with titers reduced by at most 0.58 log_10_ [18], while our previous study demonstrated that certain ultrasonic nebulizers maintained infectivity even after 60 min of operation [19]. Consistent with these findings, our results show that the vibrating mesh nebulizer preserved the infectivity of both phages tested during 10 min of use. These observations highlight vibrating mesh nebulizers as a practical, low-cost option for generating intact, active phage aerosols while avoiding the severe mechanical damage associated with jet nebulization.

The spatial mapping confirmed that the hand-held sprayer produced a highly directional aerosol stream, with phage deposition peaking along the central axis before the nozzle. At the same time, lateral spread was lower but detectable. This distribution is consistent with previous reports showing that hand-held and directional sprayers typically generate a concentrated plume with diminished side deposition [20]. Such a pattern underscores the sprayer’s suitability for rapidly targeting localized contamination hotspots. However, broader coverage would require repositioning or multiple passes.

Building on our earlier large-scale aerosolization studies in ICUs and ECMO units [6,7,8], these findings demonstrate that the hand-held sprayer provides a valuable complementary tool for localized interventions. While room-scale systems demonstrated reductions in CRAB infection rates and even complete prevention of targeted pathogens, they were constrained by practical barriers such as prolonged operation, patient relocation, and reliance on indirect outcome measures [6,7,8]. Together with prior large-scale demonstrations, these results support a two-tiered infection-control strategy: broad environmental decontamination through room-scale systems and flexible, low-cost interventions using hand-held sprayers to manage persistent or emergent hotspots in clinical settings.

Our experiments demonstrated that phage concentration and bacterial density are critical determinants of short-term surface decontamination, consistent with the density-dependent infection kinetics previously described for phage–host systems [21]. Crucially, the observed reduction in CRAB counts cannot be attributed to physical displacement or the washing effects of the aerosol spray. Three lines of evidence support this conclusion. First, we observed a dose-dependent efficacy: for a fixed spray duration, higher phage titers consistently resulted in significantly greater bacterial reduction. Second, the physical nature of the VMN aerosols precluded mechanical removal. The device generated a fine mist that rapidly evaporated or settled gently onto the surface, leaving no visible liquid accumulation or runoff that could wash bacteria off the agar plates. Third, the enumeration method accounted for displacement. Since the entire agar plate was incubated post-exposure, any viable bacteria—even if physically shifted across the surface—would still form visible colonies. Therefore, the absence of colonies confirms actual bactericidal activity rather than physical loss.

However, long-term bactericidal activity was not explained by titer alone. Persistence assays showed that ϕ2 consistently outperformed ϕ11 despite lower absolute concentrations, highlighting the importance of intrinsic biological traits such as replication capacity and environmental persistence on surfaces [21,22]. This variability underscores that phages differ markedly in their ability to provide residual surface protection, with some sustaining activity while others act more transiently. One-step growth experiments further revealed that ϕ2 (Figure S3) exhibited a shorter latent period and a larger burst size than ϕ11 (Figure S4), suggesting that differences in replication dynamics and stability on contaminated surfaces, rather than titer alone, account for its superior residual decontamination performance.

Although ϕ11 exhibited less pronounced bactericidal activity than ϕ2, as indicated by the detectable but minimal CRAB regrowth after 24 h, the residual contamination level remained extremely low—approximately 0.05 CFU/cm^2^ (equivalent to three colonies on a 9 cm plate). This value is far below the commonly accepted hospital cleanliness benchmark of 2.5 CFU/cm^2^ [23], demonstrating that even ϕ11 achieved a practically acceptable degree of surface decontamination. Moreover, post-treatment bioburden remained below this benchmark for several hours at the phage-specific effective dose, suggesting that a conservative re-application interval of around 6 h would maintain cleanliness. It should be noted, however, that this chamber study utilized single-phage preparations to establish baseline performance parameters; in clinical practice, tailored phage cocktails targeting patient-derived isolates would be necessary to achieve broader host coverage and more durable protection [6,8].

The persistence assays revealed distinct behaviors between the two phages. For ϕ2, bacterial challenge led to a slower decline in phage concentration relative to the control, indicating productive infection cycles that replenished phage numbers and prolonged surface activity. In contrast, ϕ11 showed only a modest difference between challenge and control conditions, suggesting limited amplification. This discrepancy is likely attributable to differences in replication dynamics and stability on contaminated surfaces, which are critical determinants of phage infection and decontamination performance [24]. Interestingly, ϕ11 exhibited greater overall stability on surfaces, maintaining higher titers than ϕ2 in both challenged and control conditions, yet it produced weaker bactericidal effects. This finding highlights that persistence alone does not equate to decontamination efficacy, which depends on the interplay of stability, adsorption efficiency, replication capacity, and progeny release [22]. Such variability underscores the importance of selecting phages not only for their environmental stability but also for their infection efficiency. It supports the rationale for cocktail formulations to balance these complementary traits in clinical applications.

Taken together, this study demonstrates that a low-cost hand-held sprayer based on VMN technology can effectively deliver phages for rapid, localized surface decontamination against CRAB, while maintaining phage stability and persistence under practical use conditions. The device provided targeted protection without compromising phage viability and could complement large-scale room disinfection systems by addressing localized hotspots, narrow crevices, and hard-to-reach surfaces where conventional methods may be less effective. While these findings offer preliminary evidence supporting the feasibility of hand-held phage aerosols as a flexible, cost-effective, and practical addition to infection control strategies in healthcare environments, the observations are currently limited to the specific VMN model and agar surfaces tested. Future work should therefore examine other aerosolization devices and clinically relevant materials. Crucially, we acknowledge that long-term environmental deployment of phage aerosols imposes selection pressure that could drive the emergence of phage-resistant CRAB variants. Although in vitro serial passage experiments were not performed in this study, mitigating resistance is essential for clinical translation. To address this, future implementation should incorporate phage cocktails—mixtures of phages targeting distinct bacterial receptors—and phage rotation strategies to minimize the probability of mutational escape and destabilize bacterial adaptation. Additionally, we recognize that the potential implementation of phage aerosols in healthcare settings poses safety considerations for both occupational and patient exposure. In this proof-of-concept study, our primary focus was on evaluating the physicochemical stability and decontamination efficacy of the VMN-generated phage aerosols. We did not perform specific toxicity screenings, such as testing for endotoxin levels and pyrogenicity. Furthermore, whole-genome sequencing (WGS) was not conducted to definitively rule out the presence of undesired genetic elements in the phage preparations. The absence of these specific safety profiles is a limitation of the current work.

## 4. Materials and Methods

### 4.1. Bacterial and Phage Strains and Cultivation

The carbapenem-resistant *A. baumannii* strain CRAB92040, isolated from the clinical environment, was selected as the representative clinical isolate for this study because of its broad susceptibility to 28 distinct phages, indicating high relevance and representativeness. Among these, phages ϕ2 and ϕ11 were chosen as model phages. Both belong to the top ten phages previously applied in hospital decontamination practices [6,8], and their host range ranks eighth and third among all CRAB92040-infecting phages. Therefore, combining CRAB92040 with ϕ2 and ϕ11 provides a clinically relevant and representative model for evaluating phage-based decontamination strategies.

**Figure 7:**
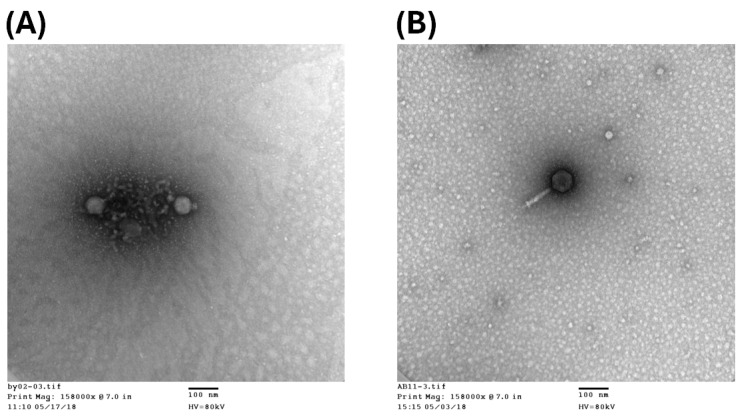
Transmission electron micrographs of the two representative bacteriophages. (**A**) Phage ϕ2, morphologically classified as a podovirus-like phage. (**B**) Phage ϕ11, morphologically classified as a myovirus-like phage. Images were taken at a direct magnification of 150,000×, and the scale bar represents 100 nm.

Transmission electron microscopy revealed podovirus ϕ2 to have a body length of 80 nm, and myovirus ϕ11 measured 230 nm (Figure 7). CRAB92040 stock cultures were preserved at −20 °C. For experimental procedures, a frozen stock was streaked onto Difco™ Luria–Bertani (LB) agar and incubated at 37 °C for 14 h for subculture. A single colony from this subculture was then inoculated into 50 mL of LB broth and incubated at 37 °C for 5.5 h with shaking, achieving a bacterial concentration of approximately 10^7^ Colony-Forming Units (CFU)/mL. The experimental work with *A. baumannii* and phage aerosols was approved by the Institutional Biosafety Committee of Tzu Chi University (Approval No. 112-009).

The phage stocks of ϕ2 and ϕ11 were initially obtained at a concentration of 10^4^ Plaque-Forming Units (PFU)/mL. For amplification, 9 mL of LB broth was combined with a 5.5 h-old host CRAB culture and the phage stock. For ϕ2, a phage stock to host bacterial culture volume ratio of 1:30 was used, followed by incubation at 37 °C for 2.5 h. Successful phage amplification was verified by the clarification of the culture when compared to a phage-free control. The resulting phage lysate was then filtered through a 0.22 μm filter to remove bacterial debris, consistently yielding ϕ2 concentrations of 10^9^ PFU/mL. For ϕ11, achieving a target concentration of 10^9^ PFU/mL for subsequent experiments required optimization of the phage-to-host bacterial culture ratio. Through testing various ratios, an optimal ratio of 1:90 with 2.5 h of incubation at 37 °C consistently produced ϕ11 concentrations of 10^9^ PFU/mL.

### 4.2. Hand-Held Sprayer and Aerosol Particle Measurement

**Figure 8:**
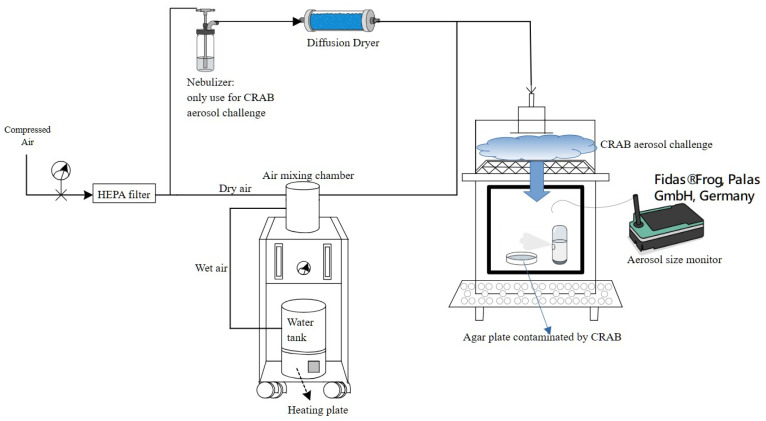
Schematic diagram of the bioaerosol test system. The test chamber was equipped with a handheld nebulizer for surface decontamination, where CRAB (carbapenem-resistant *Acinetobacter baumannii*) was spread on agar plates and subsequently exposed to phage aerosols to evaluate their bactericidal effect. An aerosol size monitor (Fidas^®^ Frog, Palas GmbH, Germany) was placed inside the chamber to continuously measure particle size distribution of the generated aerosols. In addition, a separate aerosol challenge system was established to simulate recontamination after surface decontamination. In this system, CRAB suspensions were aerosolized using a Collison Nebulizer, passed through a diffusion dryer, and introduced into the chamber to mimic airborne deposition of bacteria onto phage-pretreated surfaces, thereby assessing the residual bactericidal capacity of surface-retained phages.

A commercial NANO Mist sprayer (model W-718B) was utilized as the personal hand-held sprayer. This device operates as a vibrating mesh nebulizer, generating aerosols via a piezoelectric micropump with an oscillation frequency of 108 kHz. Its specifications include a diameter of 3.5 cm, a height of 10 cm, a liquid capacity of 30 mL, and a spray flow rate ranging from 1.25 to 1.45 mL/min (Figure 2). To characterize the aerosol particle size distribution generated by the sprayer, an air particle sizer (Fidas^®^ Frog, Palas GmbH, Karlsruhe, Germany) was used to sample the continuously emitted aerosol for 1 min (Figure 8).

### 4.3. Phage Stability in Different Solutions and Temperatures

To evaluate long-term stability, 0.1 mL of 10^9^ PFU/mL phage stock (ϕ2 or ϕ11) was diluted into 0.9 mL of either 0.9% normal saline or sterile deionized water. These solutions were stored at −20 °C, 4 °C, and 25 °C for over 300 days. Phage infectivity was monitored periodically: every 2 days during the first week, every 3 days during the second week, weekly from the third to the fourth week, bi-weekly for the following five months, and monthly for the remaining six months. A fresh aliquot was used for each measurement to avoid potential effects from repeated temperature changes. Phage concentrations (PFU/mL) were quantified in triplicate using the double-layer agar method.

### 4.4. Phage Stability Within the Hand-Held Sprayer

Phage solutions (10^7^, 10^8^, and 10^9^ PFU/mL of ϕ2 or ϕ11) were loaded into the hand-held sprayer. The sprayer was operated within an exposure chamber, and samples of the phage solution were collected directly from the sprayer reservoir at 0, 1, 3, 5, and 10 min of operation. Phage concentration at each time point was determined in triplicate using the double-layer agar method.

### 4.5. Phage Aerosol Distribution and Viability

To assess the spatial distribution and viability of aerosolized phages, a grid of 9 Petri dishes containing 3% gelatin agar was arranged in front of the hand-held sprayer within an exposure chamber. Phage solutions (10^7^, 10^8^, or 10^9^ PFU/mL of ϕ2 or ϕ11) were sprayed for 1 min. After spraying, aerosols were allowed to settle. The gelatin agar plates were then incubated at 37 °C for 10 min to liquefy the gelatin. The phage-containing gelatin solutions were serially diluted, and the number of plaque-forming units (PFU)/plate was determined by the double-layer agar method in triplicate.

### 4.6. Evaluation of Phage Spray Decontamination Efficacy on Surface CRAB

Petri dishes were prepared with initial surface concentrations of CRAB at 10^1^, 10^2^, or 10^3^ CFU/plate. For each experimental condition, a set of nine plates was arranged in a 3 × 3 grid (identical to the layout in Section 4.5) to capture spatial variability across the exposure chamber. Phage solutions (ϕ2 or ϕ11) at concentrations of 10^7^, 10^8^, or 10^9^ PFU/mL were loaded into the sprayer and applied for 1, 2, or 3 min. Three non-sprayed plates served as positive controls for bacterial growth. Following phage exposure, all nine plates were incubated at 37 °C for 14 h. Surviving bacterial colonies were then enumerated, and the survival rate was calculated based on the mean colony counts of the nine replicates relative to the control group.

### 4.7. Evaluation of Phage Persistence on Surfaces

To evaluate the persistence of phage-mediated decontamination, we investigated whether phages deposited on surfaces under optimal spraying conditions could retain their lytic activity against subsequently settled bacteria after different resting periods. The optimal spraying conditions were determined in the surface decontamination assays (Section 4.6). In this experiment, two phages were tested under their respective effective concentrations: ϕ2 at 10^7^ PFU/mL and ϕ11 at 10^8^ PFU/mL. After spraying contaminated plates loaded with 10^3^ CFU/plate of CRAB for 3 min, three plates were immediately used as controls to confirm complete bacterial elimination, while the remaining three replicate plates (*n* = 3) were left in the exposure chamber for 2, 4, 6, 8, or 24 h. At each designated time point, these plates were positioned at the central location (position 2C) to receive the CRAB aerosol challenge. This specific position was selected to ensure the plates received the most representative sedimentation of airborne CRAB, allowing for the testing of residual phage activity.

At each designated time point, the plates were challenged by exposing them to CRAB aerosols at 10^5^ CFU/m^3^ for 20 min. The aerosol challenge system is illustrated in Figure 8. Airborne CRAB was produced using a Collison three-jet nebulizer (BGI Inc., Waltham, MA, USA) operated at a flow rate of 3 L/min. The bacterial suspension in the nebulizer was prepared at 10^7^ CFU/mL. The generated CRAB aerosols were subsequently passed through a diffusion dryer, combined with a HEPA-filtered compressed air stream in the air mixing chamber (total flow rate = 50 L/min), and then introduced into the 97 L test chamber through the inlet. To ensure that each exposure reliably resulted in bacterial deposition on the plate surface, three additional blank plates were included during each run, verifying an average deposition of approximately 10^2^ CFU/plate. Aerosol concentrations within the chamber were monitored using a biosampler (SKC Inc., Connellsville, PA, USA) operating at 12.5 L/min for 20 min, and the collected samples were subsequently quantified by culture-based enumeration to verify the target aerosol density. After exposure, both phage-treated and control plates were incubated at 37 °C for 14 h, and colony counts were compared to assess how long deposited phages on surfaces could maintain their infectivity against newly settled CRAB.

### 4.8. Evaluation of Surface Phage Concentration Dynamics

To evaluate the persistence of phages on agar surfaces, sterile plates were sprayed with either phage ϕ2 at a concentration of 10^7^ PFU/mL for 3 min or phage ϕ11 at 10^8^ PFU/mL for 3 min, and the deposited phages were quantified immediately (0 h). In the control group, plates containing only phages (ϕ2 or ϕ11) were incubated to assess the natural decline of phage concentrations over time. In the test group, plates were first subjected to surface decontamination at 0 h by applying CRAB at 10^3^ CFU/plate and phage spraying under the above conditions. At 24 h, the same plates were challenged with CRAB aerosols generated from a 10^7^ CFU/mL suspension (corresponding to ~10^5^ CFU/m^3^ in air and resulting in ~10^2^ CFU/plate by sedimentation), and phage concentrations on the surfaces were further measured after an additional 14 h of incubation. Phages were recovered from plate surfaces by rinsing with 7 mL of LB broth and shaking for 10 min. The eluates were filtered through a 0.22 μm filter, and the titers were determined using the double-layer agar method in triplicate, with results back-calculated to estimate surface concentrations.

### 4.9. Statistical Analysis

The Shapiro–Wilk test was performed to assess the normality of data distribution for all experimental outcomes. One-way ANOVA was utilized to determine significant differences in phage concentrations among the nine sampled positions in the phage aerosol distribution experiments. The Kruskal–Wallis test was applied to analyze the relationship between bacterial concentration, phage concentration, and spray time on surface CRAB survival rates in the phage aerosol decontamination experiments. Additionally, for the residual bactericidal activity experiments (Figure 4 and Figure 5), the CRAB concentrations were log-transformed. For samples where no colonies were detected (N.D.), a value representing half of the limit of detection (1/2 × LOD; i.e., 0.165 CFU/plate) was assigned prior to log-transformation to facilitate statistical analysis. A two-way analysis of variance (ANOVA) was then performed to evaluate the main effects of phage treatment and time, as well as their interaction, on the decontamination efficiency against CRAB on surfaces.

For the surface phage persistence experiments, a two-way ANOVA was conducted to evaluate the effects of time (0, 24, and 38 h), treatment conditions (with or without CRAB exposure), and their interaction on log-transformed phage concentrations. Post hoc multiple comparisons were performed using Tukey’s Honest Significant Difference (HSD) test to determine significant differences between specific time points and treatment groups. All statistical analyses were conducted using IBM SPSS Statistics version 23. Graphing of results was performed using Microcal™ Origin 6.0. During the manuscript preparation, ChatGPT 4o was used for English language editing and to generate specific instrument icons (the nebulizer, personal hand-held sprayer, dryer, and aerosol monitor) used in the experimental setup diagram (Figure 8).

## Data Availability

All data supporting the findings of this study are available in the article, as well as in the Supplementary Materials.

## Supplementary Materials

The following supporting information can be downloaded at: https://www.mdpi.com/article/10.3390/antibiotics15010038/s1. Figure S1: Long-term stability of phages ϕ2 and ϕ11 under different storage conditions. Phages were stored at −20 °C (■), 4 °C (●), and 25 °C (△) in either sterile water or 0.9% normal saline, and titers were monitored for up to 300 days. (A) ϕ2 in sterile water; (B) ϕ2 in normal saline; (C) ϕ11 in sterile water; (D) ϕ11 in normal saline. Figure S2: Stability of phages ϕ2 (A) and ϕ11 (B) during aerosolization with a hand-held sprayer. Phage concentrations (PFU/mL) at three initial titers (10^7^, 10^8^, and 10^9^) are plotted against spraying time (0–10 min). Data are presented as mean values with error bars indicating standard deviations. Figure S3: One-step growth curve of phage ϕ2. PFU per infected cell is plotted over time for extracellular phage (non-chloroform, blue) and total phage (chloroform-treated, orange). An eclipse period of approximately 0–10 min and a latent period of approximately 0–15 min were observed, and the burst size calculated from the rise in extracellular titer between 15 and 20 min was ~1.97 × 10^4^ PFU per infected cell. Figure S4: One-step growth curve of phage ϕ11. PFU per infected cell is plotted over time for extracellular phage (non-chloroform, blue) and total phage (chloroform-treated, orange). An eclipse period of approximately 0–15 min and a latent period of approximately 0–15 min were observed, and the burst size calculated from the increase in extracellular titer between 20 and 30 min was ~3.2 × 10^2^ PFU per infected cell.
