# Supplementary material for: Localized Hotspot Management: Hand-Held Phage Aerosols as a Complementary Strategy for Carbapenem-Resistant Acinetobacter baumannii Infection Control in Healthcare Settings
Source: Antibiotics (Basel). 2026 Jan 1;15(1):38. doi: 10.3390/antibiotics15010038 (PMC12838338; doi:10.3390/antibiotics15010038)
Supplement: Supplementary file 1 [file antibiotics-15-00038-s001.zip › antibiotics-4044106-supplementary.pdf]

Supplementary Material：

Localized Hotspot Management: Hand-held Phage Aerosols as a Complementary Strategy for CRAB Infection Control in Healthcare Settings

Yao-Song Lin [1a], Li-Kuang Chen[1b,c], Hsiu-Yen Chien[a], Ruei-Sen Jiang[a,c] and Chun-Chieh Tseng[a*]

[a] Department and Graduate Institute of Public Health, Tzu Chi University, Hualien, Taiwan

[b] Institute of Medical Sciences, College of Medicine, Tzu Chi University, Hualien, Taiwan

[c] Department of Clinical Pathology, Buddhist Tzu Chi General Hospital, Hualien, Taiwan

[1]Yao-Song Lin and [1]Li-Kuang Chen contributed equally.

*Corresponding author

Tel. and Fax: +886-3-8574179

Email: tsengcc@mail.tcu.edu.tw

Email addresses the following:

L-YS: 109324109@gms.tcu.edu.tw
L-KC: lkc@tzuchi.com.tw
H-YC: chienhy@gms.tcu.edu.tw
R-SJ: 111324103@gms.tcu.edu.tw
C-CT: tsengcc@mail.tcu.edu.tw

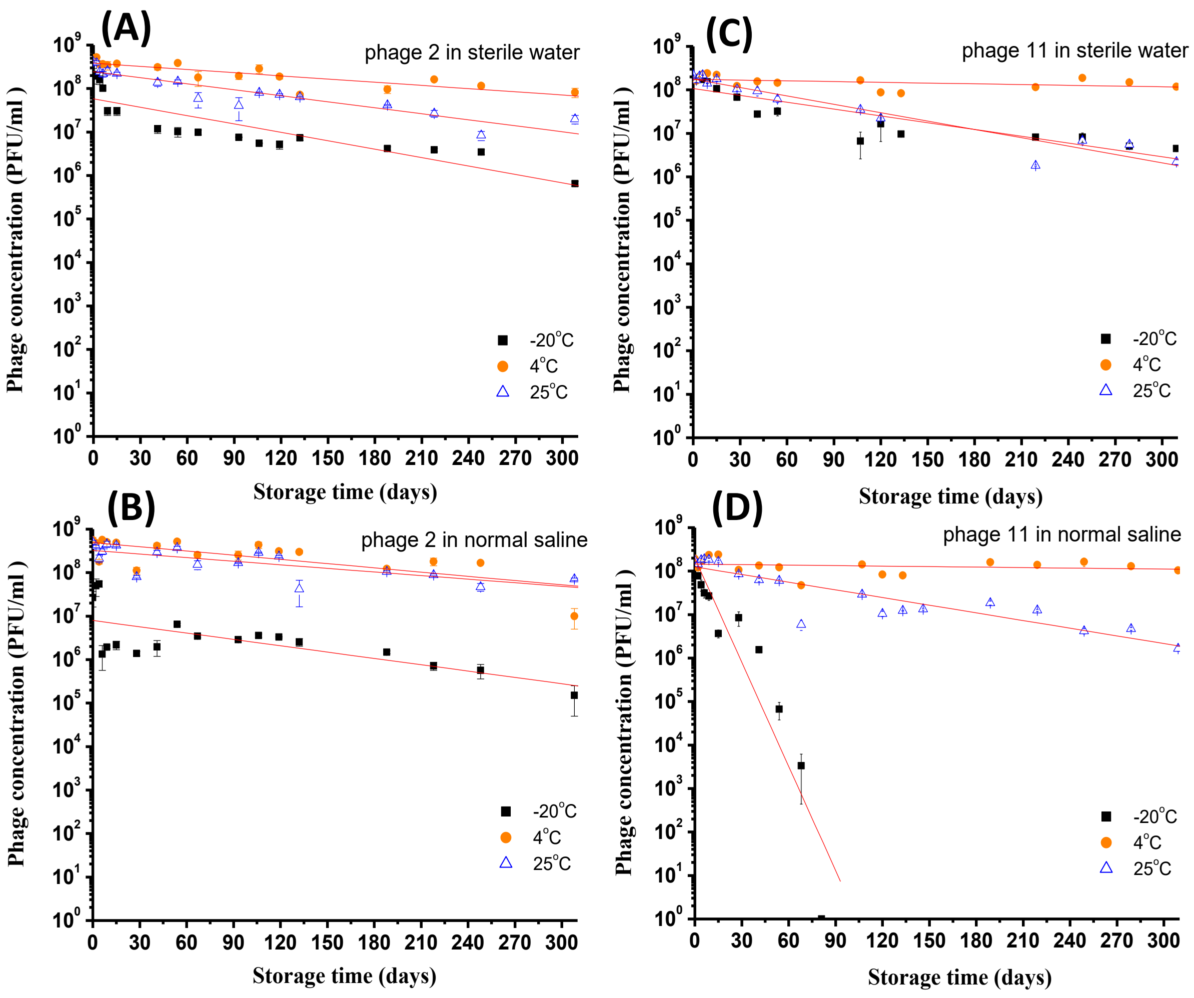

Figure S1: Long-term stability of phages ϕ2 and ϕ11 under different storage conditions. Phages were stored at −20 °C (■), 4 °C (●), and 25 °C (△) in either sterile water or 0.9% normal saline, and titers were monitored for up to 300 days. (A) ϕ2 in sterile water; (B) ϕ2 in normal saline; (C) ϕ11 in sterile water; (D) ϕ11 in normal saline.

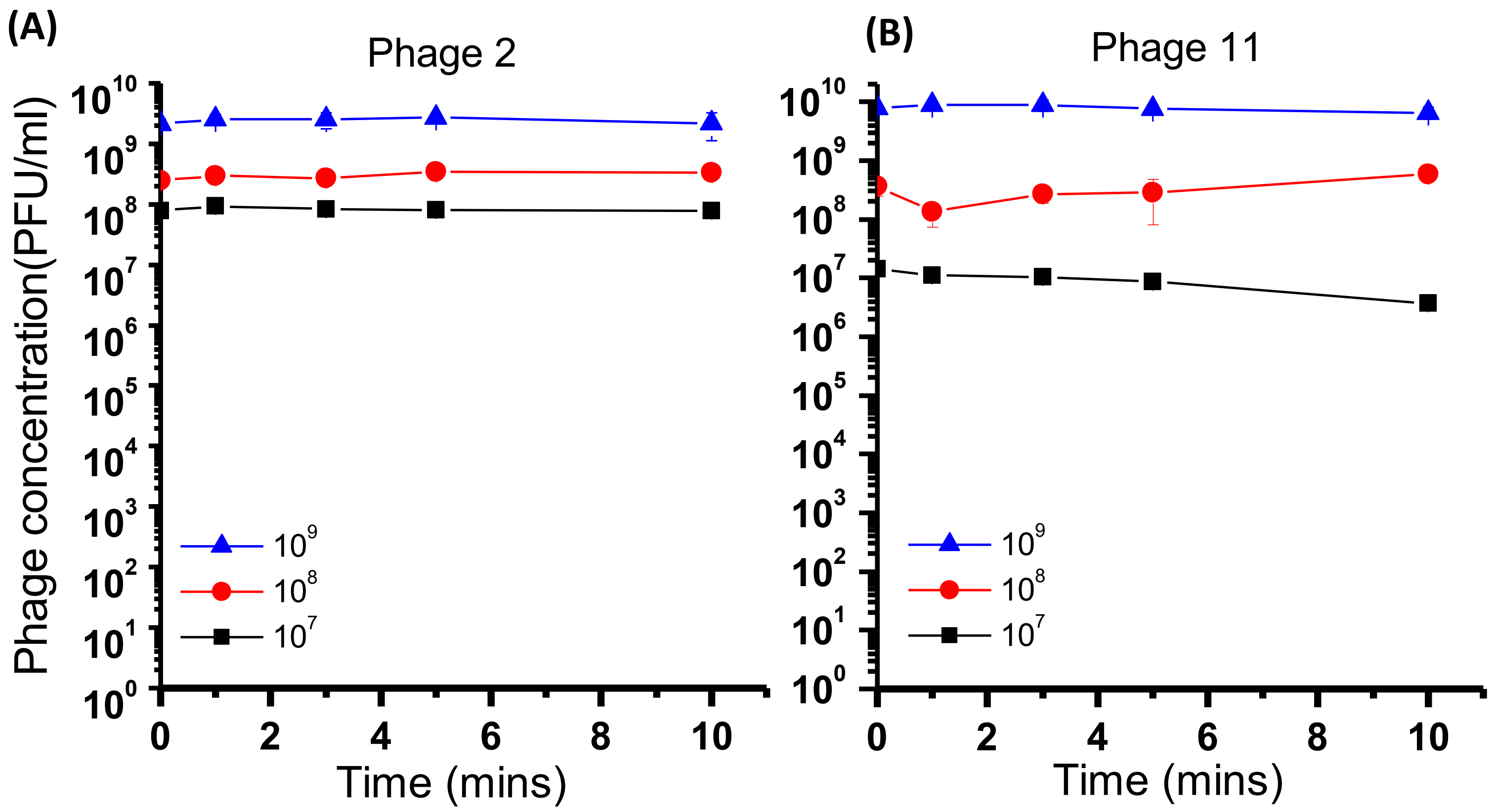

Figure S2: Stability of phages ϕ2 (A) and ϕ11 (B) during aerosolization with a hand-held sprayer. Phage concentrations (PFU/mL) at three initial titers (10^7, 10^8, and 10^9) are plotted against spraying time (0–10 min). Data are presented as mean values with error bars indicating standard deviations.

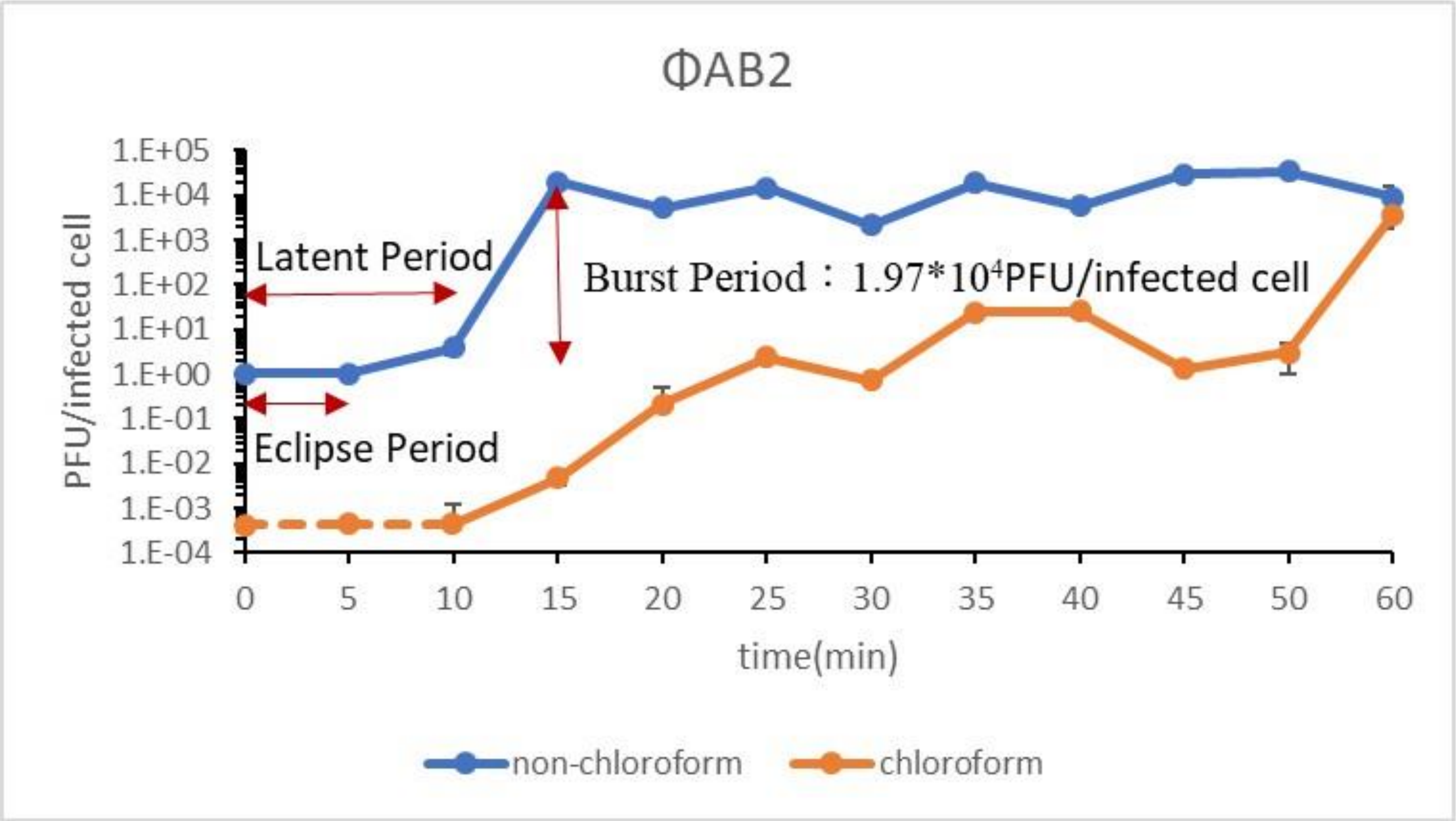

Figure S3: One-step growth curve of phage ϕ2. PFU per infected cell is plotted over time for extracellular phage (non-chloroform, blue) and total phage (chloroform-treated, orange). An eclipse period of approximately 0–10 min and a latent period of approximately 0–15 min were observed, and the burst size calculated from the rise in extracellular titer between 15 and 20 min was ~1.97×10^4 PFU per infected cell.

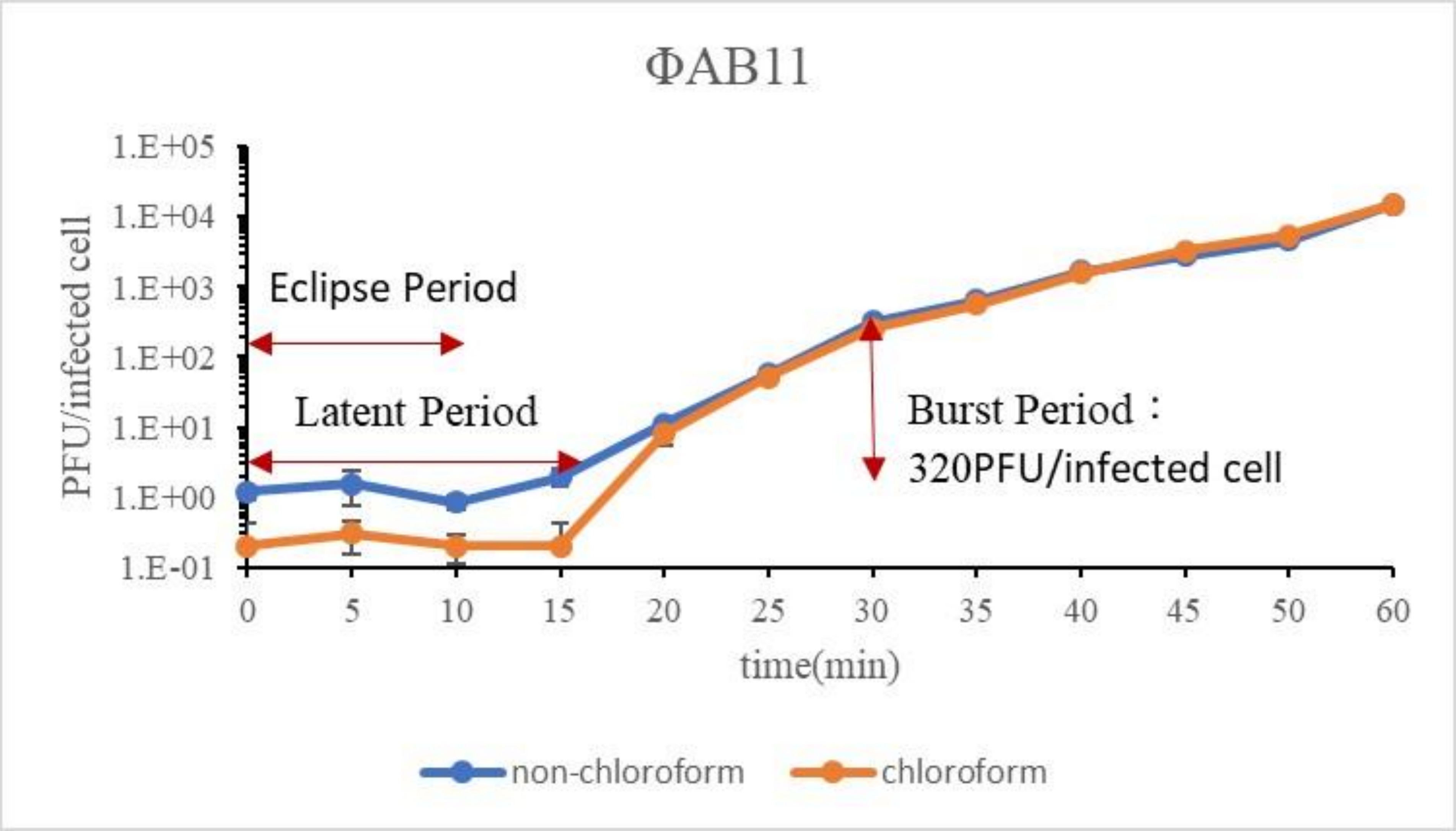

Figure S4: One-step growth curve of phage ϕ11. PFU per infected cell is plotted over time for extracellular phage (non-chloroform, blue) and total phage (chloroform-treated, orange). An eclipse period of approximately 0–15 min and a latent period of approximately 0–15 min were observed, and the burst size calculated from the increase in extracellular titer between 20 and 30 min was ~3.2×10^2 PFU per infected cell.
